# The Necessity of Monitoring Pesticide Residues in Vegetables and Fruits Using Hazard Index among Consumers

**Published:** 2019-06

**Authors:** Reza SHOKOOHI, Mohammad Taghi SAMADI, Manoochehr KARAMI, Razieh KHAMUTIAN

**Affiliations:** 1. Department of Environmental Health Engineering, School of Public Health, Hamadan University of Medical Sciences, Hamadan, Iran; 2. Modeling of Noncommunicable Diseases Research Center, Hamadan University of Medical Sciences, Hamadan, Iran

## Dear Editor-in-Chief

Nowadays pesticide residues in agricultural products have become an important challenge and a serious threat to human food security and health. Reduction of pesticides residues should be taken into consideration by public health authorities because of environmental and health problems. The harmful effects of pesticides in humans have included cancer, congenital anomalies, heart disease, Parkinson’s disease and Alzheimer’s disease (1). However the use of pesticides in the world has been rising ever since, for example, from 1960 to 2000 its consumption has been growing around 20 times, and from 2002 to 2007 has been increased 0.7 billion tons (2).

While total consumption of agricultural pesticides in developing country accounts for one-third of the world, most acute and chronic pesticide-induced diseases occur in these countries (3). According to the Statistical Centre of Iran, the total quantity of pesticide sales in 2013 amounted to close to 723596 kg and in 2014 the number of pesticide sales reached 1195269 kg (4). As reported by the Bureau of agricultural statistics, 66% of the pesticides are imported from China and India, which contain hazardous substances and impurities (5).

The conditions of the production, distribution and administration of pesticides, the high cost of legal pesticides, the poverty among most farmers and their lack of knowledge about risk of excessive exploitation of pesticides, and finally, lack of strict rules have been resulted the use of nonstandard pesticides in agricultural products (6, 7). The FAO/WHO Codex Alimentarius Commission has published a Guideline entitled “Codex Maximum Limits for Pesticide Residues” (1978) which gives both acceptable daily intake (ADI) values and maximum residue limit (MRL) values for many pesticides in raw agricultural commodities (RAC’s) from crops on which their use is approved. “ADI values are determined from animal toxicology data which given a no-observable-effect level (NOEL) and use of a safety factor. Specific MRL values are calculated from the ADI and a food factor (F), which is the decimal fraction of that RAC in the assumed typical diet” (8). One of the difficulties with measuring MRL is that dietary patterns differ substantially between countries. Calculating the risk of pesticide residues in fruits and vegetables in consumers by adults and children is characterized by hazard index (HI) which includes the concentration of pesticide residues, rate of consumption in countries or regions and consumer weight (adult or young), in order to provide more comprehensive information on the risk assessment in the communities.

If the HI is less than 1, there is no obvious risk to the population. However, if it is more than one, it is threatening for consumer health. Using this indicator, the risk of pesticides can be shown quantitatively, and managerial decisions could be made more feasible (9). In some studies, this hazard index has been calculated to assess the risk of agricultural pesticides. For example, in West Africa, the H Index for Heptachlor and Dieldrin in foodstuffs was more than 1 and warning (10). In South America, the risk of agricultural pesticides was evaluated in vegetables and the consumption of these vegetables was threatening (9).

Considering the potential health risks of contaminated vegetables and fruits, it is necessary to monitor the concentration of pesticides in vegetables and fruits periodically, and to create strict rules and regulations for proper use of pesticides. In this regard, due to various food habits and different levels of agricultural product consumption in the world, apart from measuring the concentration of pesticide residues, the HI should be evaluated.
